# On the Road to Somewhere: Commentary on Breiner et al., Matheson et al., and Palmer et al.

**DOI:** 10.1002/eat.24402

**Published:** 2025-03-10

**Authors:** Glenn Waller

**Affiliations:** ^1^ School of Psychology University of Sheffield Sheffield UK

**Keywords:** ARFID, avoidant/restrictive food intake disorder, commentary, treatment

## Abstract

This commentary considers the contribution of three papers (Breiner et al., Matheson et al., and Palmer et al.) that explore very different approaches to the treatment of Avoidant/Restrictive Food Intake Disorder (ARFID). Comparison is made with the development over time of psychological therapies for other eating disorders and how we need to be open to different possible approaches so that we can eventually find our way to the best treatment(s) in the field of ARFID. Following that, summaries of each paper are given, including consideration of their different methodologies, measures, samples, and treatment outcomes. There are also suggestions for future research that expand on the authors' ideas. The literature is clearly still very disparate, as one might expect at this early stage in the development of treatments for the range of ARFID presentations. However, these papers are all valuable pointers as to where the treatment literature on ARFID might be going in the long term.

It is important to consider the context when summarizing recent developments in the field of Avoidant/Restrictive Food Intake Disorder (ARFID). Even though the past decade has seen much greater clarity regarding diagnostic categories (particularly the “sensory sensitivity,” “fear of negative consequences,” and “loss of interest” presentations, alongside rumination and pica) and the core role of anxiety in such cases, two issues need to be addressed. First, ARFID has substantial under‐reported health and economic costs, especially given its high prevalence rate (Nicholls‐Clow et al. 2024). Second, frustration is a key experience of many people involved in treatment pathways for these eating disorders—clinicians, patients, family and service managers. Parents are often distressed at the lack of support that is available, leaving them to face the stresses alone. A lot of eating disorder services still do not have the clinical expertise to identify or treat ARFID, accompanied by a shortage of funding to do so and a tendency to focus on younger patients at the expense of adult patients. There is clearly a need for better treatments that are geared to the wider age range, so that services can incorporate them and get care to those who need it. Finally, there is discussion about where such cases should be treated (e.g., child development vs. mental health settings) and who should deliver the treatment (e.g., mental health clinicians vs. dietitians vs. eating disorder specialists). However, it is important to remember that the Institute of Medicine (2001) noted that it can take 15–20 years for new research findings to make it into routine clinical practice, so we should not be surprised at the fact that many eating disorder clinicians (and even more non‐specialists) are not fully aware of the existence of ARFID or how to address it.

As I read these three papers on the treatment of ARFID, I was reminded of a time, long ago and far away, when therapies for other eating disorders were first being developed. In those days, there were lots of ideas about what *might* work but limited evidence about what *did* work, and the spirit was one of “try it out and see whether it works.” Over time, multiple approaches have emerged as possible best candidates. For example, when the National Institute for Clinical Excellence (NICE) released its 2004 guideline, multiple therapies were seen as potentially suitable for treatment of bulimia nervosa (e.g., dialectical behavior therapy [DBT], cognitive behavior therapy [CBT], interpersonal therapy [IPT]), and anorexia nervosa was still unknown territory when it came to the best option. But things change. By the time of the revised NICE guideline in 2017, the evidence for adults had settled on CBT as the first line treatment for bulimia nervosa and the newcomer of binge‐eating disorder, but there were multiple viable (though often less effective) options for anorexia nervosa. The evidence for children and adolescents has always been more centered on family‐based interventions. Of course, we do not need to have a single “best therapy” for any disorder—if there are different routes to the same outcome, then that gives us options that we can share with the patient, making engagement more likely. And some healthy competition keeps us all on our toes, encouraging us not to accept limited outcomes but to get better in all treatment domains. It is still fun and rewarding to watch how the therapies develop, becoming more and more suitable for those patients and carers whose lives can be transformed by an effective intervention. We are definitely on the road to somewhere, but it would be a brave person who predicted too far into the future about where exactly that will be. It just has to be better than where we are right now.

Now, what does all this rambling reminiscence have to do with ARFID, and specifically with these three papers? Well, they all share some characteristics—in particular, they are all designed to help us work out what to do to treat individuals with ARFID. However, these interventions are all different approaches to that goal and at different stages in their development. When it comes to ARFID, we know that we need to get better at treatment, but we do not yet know which road(s) will lead there and which will be better for whom. Therefore, we should be looking at all promising options, just like these.

So what do these three papers contribute to answering the question of what would be best for those with diagnoses of ARFID, and how well do they do it? Let's start with Breiner et al. (2024), and their study of the acceptability, feasibility, and preliminary effectiveness of a brief training program for parents of non‐underweight children with ARFID (aged 5–12 years). The core of the program is training parents to deliver exposure‐based methods. Two training sessions might seem very brief, but if it works, then brevity and efficiency are not things that we should be objecting to. The intervention is an adaptation of a program for picky eaters, which has been piloted in order to suggest an optimum length.

First, the positives—the Breiner et al. study actually states its criteria for acceptability, feasibility, and adherence, and checks on whether they were achieved. That is to be praised, as such concrete prediction is far less common than it should be in the research literature. Furthermore, qualitative and quantitative outcomes were considered. So did the intervention work? There were indeed some positive outcomes (including additional foods incorporated into the child's diet). On the negative side: there were a lot of missing data, meaning that some analyses could not be conducted; retention was poor; those parents who stayed with the program were far more likely to request a booster session than the pilot work had suggested; and adherence was limited.

To my eyes, this was a study with mixed qualities—some parts were weak, some were good, but the good parts were worthy of reporting for clinicians and researchers reading this special edition. The future challenge for this intervention is going to be primarily what the authors identify in their Discussion—how can this promising approach be adapted to make it more feasible, increasing adherence, retention, and effectiveness? The authors make some general suggestions, but I would like to have seen the qualitative study conducted more usefully. Instead of only interviewing those who completed the intervention, I really wanted to hear from those families who did not complete the therapy, to find out why they did not make it to the end. I was not sure about the suggested use of the ARFID‐PTP as a waitlist intervention, given its poor retention rate, but I would not rule it out if that problem can be solved.

Turning to Matheson et al. (2024), we see a similar age group (6–12 years), but these patients were all underweight and were far more likely to be female than those in the Breiner et al. study. Here, the intervention is much longer (14 sessions) and targeted separately at the individual with ARFID and at parents. The focus was on psychoeducational and motivational intervention strategies.

The outcomes were more positive than those of Breiner et al. in terms of the parents' ratings on the Pica, ARFID, and Rumination Disorder Interview (PARDI; Bryant‐Waugh et al. 2019), as well as retention in the study. However, there was minimal impact on expected body weight. Furthermore, the measure of parental self‐efficacy showed limited change (and the psychometrics of the measure suggested that it should not be relied on).

Thinking of future developments of this research, I wish that we had been given some indication of whether there had been improvements in motivation and learning, given that these were the foci of the intervention (rather than any focus on changes in cognition or behaviors). I would also like to have seen information about changes in the range of foods eaten. Finally, I wonder whether a “primarily play‐based” intervention is as useful for 12‐year‐olds as it is for 6‐year‐olds. All questions for future consideration as this approach is rolled out further.

Finally, Palmer et al. (2024) present work that is further along the path than the other two papers. CBT for ARFID (CBT‐AR) is already at the stage where the pilot evidence is taking us toward clinically useful conclusions about its adequacy for adolescents and adults. So, we already know the likely outcome of CBT‐AR. However, this case series is large enough to let the authors address the critical question of what predicts who will do well in therapy. The retention rate was reasonable, the gender split was close to even, there were underweight and non‐underweight participants, and the age range was wider than in the other two studies, including adolescents and adults.

First, Palmer et al. show that those with ARFID who fear aversive consequences of eating are the people who do best in CBT‐AR. Second, nothing else was linked to subsequent outcomes. Nothing at all. All possible predictors were reasonable hypotheses (e.g., age and weight), but none of them predicted better or poorer outcomes. While the authors seem most interested in the positive result, I do not entirely agree. I would say that the negative results are equally important. If a characteristic like age or weight does not get in the way of therapy, that is one fewer thing that we have to focus on, select by, or adapt therapy for—all making the delivery of the therapy far more straightforward. That is the case with CBT for other eating disorders (CBT‐ED), where very few predictors have been found outside of the accurate delivery of the therapy. While that lack of predictors might surprise many clinicians, if we do not need to make adjustments for duration, severity, gender, etc. (e.g., Radunz et al. 2020), then that is no bad thing.

My one disappointment with Palmer et al.'s study is that it does not consider the one factor that we know is a strong predictor of outcome in CBT‐ED and in CBT more widely—early change (e.g., Beard and Delgadillo 2019). So, if you have the necessary data, how about a little retrospective analysis, Palmer and co., and tell us whether it is as important to push for substantive change from the beginning? That would be a real benefit in the training and supervision of clinicians who are going to be working with ARFID in the future.

## 1 Conclusion

Overall, then, these are approaches that differ in their hypothesized active agents, cover a range of ages, and differ in their gender balance. So they cannot be compared as being like‐for‐like. All three studies are US‐based, which means that the work of teams in the UK, the Netherlands, Australia, and elsewhere is not represented here. I suspect that the shift in the balance of such research to the US over the past decade or more has a lot to do with the availability of research funding there since ARFID entered DSM‐5. However, there are excellent teams working elsewhere on similar and different approaches to these disorders, so who can say which approach(es) will prove to be the ones that we embrace in the future for their evidence and deliverability.

And let us also remember that there is a clear need for research of the sort that these three teams are pioneering. Even taking a cautious approach to diagnosis, meta‐analysis suggests a point prevalence rate of ARFID of 4.51% (Nicholls‐Clow et al. 2024), with relatively similar numbers between males and females and between adults and children. Maybe the problem of ARFID does not just go away when the patient hits young adulthood, as I remember being the legend when working with such cases early in my career. The prevalence of ARFID is clearly very large compared to, say, anorexia nervosa. Given the scope of this problem, we very certainly need all the innovative research that we can get to support us in working with this population and their parents. Those with ARFID and their loved ones have suffered from a degree of neglect for far too long.

Yes, we need larger samples, full randomized controlled trials, longitudinal studies, and much more, but given the relative youth of the construct of ARFID, we are not doing badly. It has been good to see the treatment of ARFID being taken more seriously and systematically in the past decade, and these three studies show how far we have come (as well as how far we have to go). They show that we are on the road to somewhere, but it would be a braver person than me to say what we will be treating as “conventional wisdom” when the next NICE guideline is published. So, no favorites here—these teams all deserve plaudits for what they have done in taking the field further. What comes next from these authors and other teams promises to take us further forward. Whatever route that road might take, it has to end up with the destination of better treatment outcomes across the spectrum of ARFID presentations.

## Author Contributions

**Glenn Waller:** conceptualization, writing – original draft, writing – review and editing.

## Conflicts of Interest

The author declares no conflicts of interest.

## Data Availability

Data sharing is not applicable to this article as no new data were created or analyzed in this study.
